# Persuasion, Adaptation, and Double Identity: Qualitative Study on the Psychological Impact of a Screen-Detected Colorectal Cancer Diagnosis

**DOI:** 10.1155/2018/1275329

**Published:** 2018-06-07

**Authors:** Lesley M. McGregor, Sara Tookey, Rosalind Raine, Christian von Wagner, Georgia Black

**Affiliations:** ^1^Department of Behavioural Science and Health, University College London, London WC1E 6BT, UK; ^2^Department of Applied Health Research, University College London, London WC1E 6BT, UK

## Abstract

The NHS Bowel Cancer Screening Programme (BCSP) is aimed at reducing colorectal cancer (CRC) mortality through early detection within a healthy population. This study explores how 5 people (three females) experience and make sense of their screen-detected diagnosis and the psychological implications of this diagnostic pathway. A biographical narrative interview method was used, and transcripts were analysed using a thematic analysis with a phenomenological lens. Themes specifically relating to posttreatment experience and reflections are reported here: Do it: being living proof, Resisting the threat of recurrence, Rationalising bodily change, and Continuing life—“carrying on normally.” Participants described their gratefulness to the BCSP, motivating a strong desire to persuade others to be screened. Furthermore, participants professed a duality of experience categorised by the normalisation of life after diagnosis and treatment and an identification of strength post cancer, as well as a difficulty adjusting to the new changes in life and a contrasting identity of frailty. Understanding both the long- and short-term impacts of a CRC diagnosis through screening is instrumental to the optimisation of support for patients. The results perhaps highlight a particular target for psychological distress reduction, which could reduce the direct and indirect cost of cancer to the patient.

## 1. Introduction

Colorectal cancer (CRC) is the second most common cause of cancer death in the UK; nevertheless, it is also one of the most treatable cancers if detected early [1–3]. In 2006, the English National Health Service (NHS) introduced a population-based screening programme to help reduce CRC mortality. The Bowel Cancer Screening Programme (BCSP) invites men and women aged 60–74, and registered with a general practice in England, to complete a guaiac faecal occult blood test (gFOBt) at home, every two years. The gFOBt involves the individual collecting small samples of their faeces from three separate bowel movements and sending the completed test kit to their local screening hub, where the samples are tested for traces of blood. Blood in a stool sample could be a sign of bowel cancer and so, if present, a follow-up endoscopic test (i.e., colonoscopy) is offered to determine the cause of the bleed and to initiate the necessary treatment [4]. A Cochrane review by Hewitson and colleagues found that taking part in gFOBt screening can reduce CRC mortality by up to 25% [5].

Uptake of the BCSP is a major public health concern with only 59% of eligible invitees completing and returning a gFOBt [6]. Much research has focused on identifying the psychological and practical barriers to test completion, so as to understand the reasons for non-participation and to tailor interventions accordingly. Reasons for non-test completion include a fear of cancer and feeling healthy at the time of the screening invitation, or having a belief that the process of the test (e.g., sampling faeces and storing faecal samples) was unsanitary or embarrassing [7–10].

There has been additional interest in the psychological impact of CRC screening participation (from receiving the invitation and test materials in the post to receiving an abnormal test result) with predominantly quantitative studies conducted and a negative impact only found in the short term [11–16]. What remains missing from the literature is a qualitative exploration of the impact of being diagnosed with screen-detected CRC.

Qualitative research is available that explores the experience and impact of investigative tests via the NHS referral process in England [17, 18]. Both studies interviewed patients who were referred via their general practitioner (GP) or through the accident and emergency department when CRC was suspected, and both indicated a period of heightened anxiety and a need for more information within this pathway to help reduce fears and uncertainty at this time. Individuals interviewed within Worster and Holmes' study had been diagnosed with CRC and had recently received therapeutic surgery [17]. Further analysis of their interviews included themes of continued fear and anxiety at the realisation of their cancer diagnosis and prognosis [19]. Unlike CRC screening, patients are referred for tests after presenting with symptoms and so their experience may be different to those tested and then diagnosed as part of a screening pathway, particularly given the effectiveness of the BCSP and the focus on early detection.

While we do not know how a diagnosis initiated through completion of the gFOBt impacts individuals, interesting work has been done in the UK using an alternative screening methodology. Miles and colleagues conducted an interview study with 24 people diagnosed with CRC following participation in a trial using flexible sigmoidoscopy (FS) as a screening procedure [20]. Thematic analysis of the interview transcripts indicated a general feeling of shock following the diagnosis, which was an unexpected outcome given a lack of perceived symptoms. Interviewees had taken part in screening as a way to reassure themselves of their good health as opposed to being a means of confirming disease. Their initial reaction was one of fear, which was later diffused by an understanding and appreciation that the screening had caught the cancer at an early, treatable stage.

A review by Aziz concluded a need for further research into cancer survivorship by suggesting that individuals experience positive and negative acute and long-term effects of cancer (psychological, physiological, and social), which occur as a direct response of a cancer diagnosis [21]. Such effects can include identity reconstruction and an integration of the cancer experience into one's self-concept [22] and how one views themselves and their body [23]. There is currently no research considering cancer survivorship specifically in relation to patients diagnosed through the UK screening programme, and yet as the programme continues, more and more people will be diagnosed early and become such survivors.

In this study, we aimed to better understand the experiences associated with receiving a cancer diagnosis within the screening programme, as described by survivors, to identify their needs and adapt the support that has been provided to patients diagnosed symptomatically. To do this, we review the events, descriptions, and reflections encased within the individual, personal stories of those previously diagnosed with CRC through the NHS BCSP.

## 2. Materials and Methods

### 2.1. Study Design

A phenomenological lens was applied to explore the experiences of participants in an ideographic and inductive way. This means that we view the screening, detection, and treatment of cancer as an experiential phenomenon within a broader context of constant social interactions and bodily sensations (the *lifeworld*) [24]. We also take as a principle that people are constantly interpreting life post hoc, both alone and in the interview context.

### 2.2. Recruitment and Participants

Participants were initially recruited to take part in a study where the aim was to develop and evaluate a narrative-based leaflet that would help engage future CRC screening invitees and address some of the key barriers to uptake (e.g., feeling well) [25, 26]. For the development of the leaflet, 21 individuals who had previously taken part in the NHS BCSP were recruited and interviewed about their screening experience [25]. A CRC diagnosis was not part of the eligibility criteria; however, for the current study, only the personal stories of those who were diagnosed with CRC through the screening programme were considered for analysis. Although 8 of the 21 interviewees had had a diagnosis of cancer following screening, only 5 of the interview transcripts were of a depth and quality suitable for analysis. All 5 interviewees consented for their interviews to be analysed in this way.  Table 1 provides characteristics of the 5 participants whose stories were analysed for this study.

All participants included in this study were recruited via the UK-based charity, Beating Bowel Cancer (BBC). A recruitment advertisement for the study was published on the charity's website and corresponding Facebook page. Emails containing details of the study were also sent directly from a BBC representative to charity members known to have been diagnosed with CRC through the NHS BCSP. All interested individuals were asked to contact the researcher (LM) to confirm their interest and discuss possible participation. A patient information sheet and consent form were sent to those interested, for further consideration, with a subsequent telephone call confirming receipt, and, where relevant, the date and time for the interview was arranged.

### 2.3. Procedure

All interviews were conducted in a venue and format convenient for the participant: home interview via telephone (*n* = 4) and face-to-face interview within the university (*n* = 1). The author LM conducted all interviews except one telephone interview, which was carried out by a trained MSc Health Psychology student. Telephone interviews have been identified as a successful alternative to face-to-face interviews, particularly where sensitive topics are included, and the participants would otherwise not be included for geographical reasons [27].

Interviews were conducted following a narrative interview design that is aimed at allowing events and thoughts to be described in the participants' own words and in the order that came most naturally to them. The interview design had 3 stages. The first involved the following “Single Question aimed at Inducing Narrative” (SQUIN) [28]:

*Can you please tell me your story of how you came to do the bowel cancer screening test kit and how it all turned out? Tell me the events and experiences which were important for you. Begin wherever you like. Take all the time you need.*

The participant's response to this question was uninterrupted; only reiterations of the main question and encouragement to continue were offered from the interviewer when necessary. When the participant indicated that they had come to the end of their story (e.g., “And that is basically my story.”), the interviewer then proceeded to the next stage whereby more probing, open questions were asked to gain further information and clarification to the initial, relevant “story” content. These questions were similar to Wengraf's “Topic Question aimed at Inducing Narrative” (TQUINs) and followed the order of the issues and statements made in the initial story provided so as to follow the participant's line of thinking [28]. The final stage then involved asking predetermined questions relevant to the aim of the research that had not been covered in the first two steps, for example, “What advice would you give someone who has just received the gFOBt kit in the post?” Interviews lasted between 40 and 120 minutes and were conducted between the 7 October 2011 and 9 November 2011. Ethical approval was granted by University College London's Research Ethics Committee (letter dated 20 June 2011).

### 2.4. Data Analysis

A thematic analysis was applied to the interview transcripts [29, 30]. First, each transcript was read several times for familiarity and annotated. Sections of the text were highlighted and labelled in a free coding process, with codes pertaining to a participant's meanings and experiences. Once this process had been completed for each participant, codes were analysed between and across cases and developed into interpretative themes.

### 2.5. Quality Assurance

Prior to analysis, interviews were audio-recorded and transcribed verbatim. Each participant was offered the opportunity to proofread their transcript in order to confirm the content. Only two were returned with minor grammatical changes or “unclear” words completed; no substantial changes to the content were made. The final transcripts were then analysed (led by GB with the support from LM and ST). To ensure quality and rigour of the analysis, the themes were revised by iterations of analysis with the transcriptions and repeated discussions with the other authors during the initial thematisation and interpretative thematic processes. Themes were reorganised and renamed to ensure appropriate representation of the data, and a historical paper trail was kept detailing all stages of the analysis.

## 3. Results

A number of themes were identified from the narratives analysed that have not been reported here, including “shock at the diagnosis” and “a need to regain control of the situation at the point of diagnosis,” mirroring those found in an earlier study [20]. For this article, we have elected to report on the themes found in relation to the current position of survivorship due to the gap in the literature on this topic. Our analysis identified four themes: Do it: being living proof, Resisting the threat of recurrence, Rationalising bodily change, and Continuing life—“carrying on normally.” Together, these themes describe how participants now make sense of their cancer diagnosis and have adjusted to life afterwards.

### 3.1. Do It: Being Living Proof

Having experienced the initial shock of diagnosis, participants expressed passionate feelings about gFOBt completion, considering it to be something which had saved their lives: this we understood from their primary expression of feeling “lucky.” They felt highly motivated to persuade other people to carry out the test. We will start with Simon who espouses this idea most fully. As a proactive bowel cancer charity member and a confident speaker, this quote illustrates his fervent appreciation for what the gFOBt did for him and the necessity of blindly complying with it:

*For me, if you get the kit it's a no brainer, don't even think about, just do it. […] I consider myself to be very lucky and that decision I made to complete that screening kit in December 2008 was probably the best decision I've ever made in my life. (Simon)*

Simon is persuading us with two notions that the evidence surpasses criticism (a “no brainer”) and, furthermore, that he is lucky to have been diagnosed with cancer through screening. Other participants echoed these feelings, particularly seeing the test as something which would catch cancer early and accurately. Bill offers a scientific basis for taking part—the test is accurate, and the test will see if there is “something there.”

*Do it, do it. I mean, you know, it, it could save your life. You're stupid, if you don't… Do it. Erm, because it's an opportunity, it's an opportunity that people 20 years ago didn't have. You are now being given the opportunity to check once and for all, ‘cause it is pretty accurate, check once and for all that erm, at least there is something there. (Bill)*

So again we witness the emphasis on luck, this time expressed as fortunate “opportunity,” and an implied criticism of people who might choose not to take the test. Again, based on the inherent righteousness of their own near miss, Celia and Bill are incredulous. To avoid the test would be “silly” or “stupid”:

*So I mean I think if anybody had the test and didn't do it I think they're very silly. You know erm, because even if you go oh that's not going to happen to me and erm, you know, and it could do, as I proved. (Celia)*

Like Simon, Pauline felt the need to persuade others to take the test without consideration, information, or hesitation and to believe in the rightness of doing so.

*Get on with it. Don't eh, this is... I'm being very naughty here. Don't… Just, just do it. In your time do it, but make sure you do it within the time you need to have done it and send it off and say some prayers, you know, or ... but make sure you get it done and sent off. (Pauline)*

Another persuasive force in the imperative to persuade others is expressed as “catching it early.” Helen uses her own example, despite being diagnosed with secondary tumours:

*So I said “You do it”, and I got onto everybody now that I know, do the test whenever you get it because the importance of catching it early. I mean I caught mine really as early as, you know, the gaps between the tests and yet it has spread to my lymph nodes and it has gone to my lung, so if I hadn't have done that test and I'd have just left it, it could have been too late, you know, and so if everybody does the test as soon as they get it they've got a much better chance… (Helen)*

Helen is so enthused here that she wants to impart her new wisdom to “everybody now.” Simon and Helen both make sense of their own cancer diagnosis as direct proof of the need for others to follow their example:

*I'd tell them for Christ's sake do it, based on my own experience (Simon)*

*I just say to people ‘Do it', the National Health are not sending it out for fun, it's for your benefit, and my boss, my ex-boss, actually had afternoon tea with him yesterday, we're still in touch, he said he'd never done it, he said “I didn't like the thought of doing it”, he said, “But now I've heard your story” he said “I'd do it”. (Helen)*

All participants are mindful of their own luck in being diagnosed and express a desire to rebalance their good fortune and give something back by persuading others to participate in the screening.

### 3.2. Resisting the Threat of Recurrence

Having been diagnosed with cancer when they were ostensibly symptom-free had created a feeling of unease and distrust in bodily symptoms. The potential for, or reality of, cancer recurrence dominated their thinking, and yet they wanted to break free from it and be “positive.” Pauline tried to manage her fears about watching and waiting but admitted she did think about it:

*People are still aware, if you've had cancer, it's still around […] I don't know whether it's, it's not what you should think, but it's, you know, you should get on with what you've got to get on with, really, and [Mmm] as far as I'm concerned at the moment. I, I can't say I don't think about eh, if it's still lurking around, but [Mmm] I, I don't think we've got to think about it. I think we've got to be positive. (Pauline)*

Simon was additionally diagnosed with prostate cancer during investigations for his colorectal cancer and has to undergo regular testing. Still, he was able to dwell on the lack of bone metastases as a reason to stay positive:

*…but even on the prostate I'm only being seen every three months, prostate cancer normally goes to your bones. I've recently had a full body bone scan and I'm perfectly clear, so I guess that's me and now leading the same active life that I had before…*

Part of staying positive was to illuminate the unknown by having further tests and then “dealing with it,” suggesting that whatever the test revealed was less frightening than the imagined horror of the creeping and lurking disease. Testing had particular power as a way of knowing what was happening inside but also imposed a timeframe within the continuum of existence. The only ally against the unknown inside the body was a CT or MRI scan—time between scans was defined as “waiting.” Celia was the main proponent of this idea:

*I tell everybody at work, I just live 6 months at a time. I have the scan, they tell me I'm okay so therefore I know I've got, I should be alive for the next 6 months, end of. (Celia)*

Celia is no longer able to imagine being “okay” beyond the 6-month timeframe between tests. She has lost the ability to “detect” her own wellness and is left dependent upon machines and tests to provide her with a future.

### 3.3. Rationalising Bodily Change

Part of the new horizon of life after diagnosis and treatment for all cancer patients is the adjustment to changes in one's body and the way in which they perceive their illness in relation to their new embodied self. We also realised that having been screened from the age of 60, other people of the same age would be adjusting to various other changes, such as retirement and physical capabilities. Therefore, diagnosis of cancer through the screening programme (and related bodily treatment, recovery, and long-term adjustment) may be related to wider experiences of change. After being diagnosed through screening, all participants mentioned that their bodies had changed, provoking them to isolate and evaluate their bodies as objects, relative to other potential states, such as the time before cancer diagnosis or an imagined state of much worse illness. Helen admitted that she was reluctant to see herself as frail:

*Erm, this was a serious illness and I think just admitting frailty, which I don't like doing, {laughs} you know, and I had to admit that, you know. (Helen)*

There was a need to project wellness and normality, which may be due to societal expectations and media narratives around positive thinking with respect to cancer. Thinking about the difficult changes their body had gone through while trying to project positivity suggests a separation of body and self. Bill inhabits his body with a feeling of pride, particularly in relation to medical performance. Throughout his transcript, he described his body in two ways, with his “good” body commended for its physical performance in spite of his “bad,” tired, and ill body. He champions his response to chemotherapy and blood tests:

*I had absolutely no side effects at all apart from err, a taste of aluminium in my mouth all the time. Erm and the degree, I suppose a degree of tiredness. So in fact I swanned through chemotherapy without any major problems and since then I've met people who've had far more problems than me. (Bill)*

Other participants were not so much proud as tolerant of bodily change. Simon was keen to focus on the resumption of his normal life, going on trips and out to restaurants. This was then qualified by the need to take loperamide and to be “surreptitious” about who he shares a room with in case his symptoms embarrass him. However, the focus was very much on returning to life as it was “before,” framing his desired life in the time before diagnosis:

*so erm I guess that's me and now erm leading the same active life that I had before, apart from the occasional embarrassment with diarrhoea and that sort of thing.. (Simon)*

In a more regretful account, Pauline yearns for her body to resume its pre-cancer form, saying “*I just wanted it, again, I wanted to be like I was before this all started to happen*.” Yet in another extract, we can see that she also wants to adjust:

*You, you learn to adjust. You just adjust accordingly, [Yeah] so it's eh, em, you know, I can't sort of dance and do quite, I can, but not quite [Mmm] so, so much, but, you know, as you're getting older it's less important. What's important is that you're alive and you're well and you're able to do a certain amount of things. This is the thing. (Pauline)*

These extracts point to an experience of bodily change which provokes a consideration of the body as separate to self. As the body has become less able or changed, participants evaluate their ability to do everyday activities, especially those enjoyed before illness. Feelings about bodily change and bodily performance are evaluated relative to other known cancer patients, to former activities and abilities, and to the alternative of severe illness and death.

### 3.4. Continuing Life—“Carrying on Normally”

Linked to the previous theme about the need to portray and embody positivity, the participants made sense of being diagnosed with cancer through screening by using metaphors to show themselves to be strong or normal. The ability to continue effectively with life was always related to adopting a well persona; any worry about frailty or morbidity was rejected or avoided.

The gravity of cancer as their illness provoked strong metaphors from the participants about their ways of achieving a positive outlook. Here, Simon describes the importance of his granddaughter's account of him being invincible, where his cancer is portrayed as a meteorite:

*She said “I'm just picturing you granddad”, I said go on, she said “The world has just been struck by a meteorite, it's about to be destroyed and you're standing on the top of this hill and you're looking down on the devastation covered in dust and you're dusting yourself off and saying ‘is that the best you can throw at me', and that has stuck with me forever and I go back to it. (Simon)*

The cancer is obviously felt to be an event of overwhelming might and strength both for the granddaughter and for Simon in his adherence to her description. Simon is comforted by this image of him, banishing his own depressed feelings and vulnerability. Celia describes a private reaction to the cancer experience which is expressed as “shouting and screaming,” but like Simon, she feels that inhabiting the positive identity does her more good:

*I mean I can shout and scream with the best of them, and that when I'm indoors on my own but it's not going to do any good is it. So err, you might as well put a grin on your face and go out there and live with it. You've got it so, you know, deal with it. (Celia)*

Helen seems to be at a different point in the process to the other participants. She recognises herself as a shadow, but this has as much to do with cancer as with her advancing age and her retirement:

*I just became a shadow of my former self and that was it, you know, mentally and physically. It was, in a way a sense of loss, the whole thing, because I left work finally in the April… I was also chair of governors at the school and had to give that up because I knew if I was going to have operations and chemo and radiotherapy I wouldn't be able to fulfil my duties there. (Helen)*

Despite these feelings, Helen also describes a need for normality, using the drama of a “howling wind” to exemplify her resistance and strength:

*Erm, it's changed a bit but, I'm doing, I'm going to a firework party on Friday, I'm starting sorting out things for Christmas, you know, went down the beach yesterday, despite the howling wind and everything, and sat behind the shack and had an ice-cream with the son and the grandchildren, you know, so, you know, you just carry on normally.*

We notice here that “carrying on” and “dealing with it” are the ultimate goals for these participants. Metaphors for the might of cancer are used to impress upon us what they have faced: images of strength are invoked in an effort to navigate a course of uncertainty and emotional difficulty, but they must keep moving.

## 4. Discussion

The aims of the present study were to explore how people experience and make sense of their screen-detected diagnosis of CRC and the psychological implications of this diagnostic pathway. While participants described their experience from receipt of the test to their present, posttreatment self, in this paper, we focused on the novel themes that describe the experiences after treatment.

Cancer survivorship is defined as the acute and long-term, positive and negative, physical and psychosocial effects of a cancer diagnosis and treatment [21]. Screening increases the likelihood of a cancer being found at an early stage and of survival. Therefore, those diagnosed by screening are likely to enter into stages of survivorship where effects of the initial diagnosis can continue long into their lifetime. Previous research suggested that the extent to which individuals identify with their cancer experience may be an important aspect of their adjustment to longer-term cancer survivorship [22, 31] and how one views themselves and their body [23].

For our participants diagnosed via screening, once they entered into a stage of survivorship, they attempted to assume the role of the well person, which involved the construction of two different identities: a strong, healthy, humorous, and public/social identity, and a diseased, frail, embarrassed, and child-like identity. In order to continue with life after illness and treatment, one had to assume a new identity, which was different from the preillness and illness identities. Taking on the strong, almost invincible role was important as it allowed people to “just get on with it.” Nevertheless, participants acknowledged that often taking on the new “well role” meant that they felt great loss for their former identities (e.g., Helen's loss of her identity as a professional woman), leaving them a mere “shadow” of their former selves. Yet despite their difficult transitions, participants continued on in life, with the belief that one should “just carry on normally, despite the howling wind and everything.”

Similar to Miles and colleagues, participants described feeling shocked at their diagnosis as they believed themselves to be well [20]. Their pre-screening illness representation meant that the decision to complete the test was led by a desire for verification of wellness or out of responsibility and support for the NHS. As time went on, these feelings of shock were later diffused and replaced with unequivocal gratitude for the opportunity to catch the cancer early and to reach a stage of “survivorship.” Given this context and despite the identity adjustment described above, participants seem to be driven to encourage others to do the same with the message that they too can “catch it early” and be saved. To date, research has not looked at the relationship between screen-detected CRC diagnoses or survivorship and attitudes toward (public promotion of) related screening services. This is important given the survivor's narratives surrounding screening in the public sphere and knowing that someone with cancer can often influence the decision of others invited to take part in screening [8, 32]. Future work to further explore the underlying motivations for the almost evangelical promotion of CRC screening by survivors, as highlighted in this study, and how it compares to those with other screen-detected cancers (i.e., breast or cervical) and those who were diagnosed with CRC out with screening, would also be beneficial to inform interventions for either survivorship support or screening promotion.

Participants expressed simultaneously contradictory emotional responses to their diagnosis and the continued retesting for cancer, suggesting that they experienced both fear and relief at multiple stages throughout their adjustment to life after diagnosis and treatment. This adjustment included the formation of a new relationship with their bodies, one which incorporated dual identities of an embodied strength and resilience as well as frailty and constant reappraisal of their health and adjustment to the new norms of their body. Chronic health issues experienced as a result of cancer treatment facilitated a feeling of unease and mistrust of one's body. Participants described this mistrust of their body and a mistrust of their ability to assess their own health when they expressed the conflict over experiencing a body that previously *felt* well [8] but was *actually* inhabited by an “insidious cancer that crept within.” Their new embodied experiences include assimilation of the new and embarrassing symptoms, admittance of weakness, and creation of a new definition of what is “normal” for their changed bodies.

While much of the current literature in the area has focused on identifying the psychological and practical barriers to test completion [7–10, 33] and the psychological impact of CRC screening in general [11–16], most studies have been quantitative in nature and have not looked into the experience of and psychological impact of screening-detected CRC diagnosis in a longer term. This paper therefore offers an important addition to the literature to date, allowing an insight into the long-term consequences of a screen-detected CRC diagnosis. Future work could consider comparisons with those who reach a similar stage following a CRC diagnosis from symptomatic or emergency presentation to allow a more multidimensional understanding of CRC survivorship. Indeed, in a recent quantitative survey study, differences in patient-reported care experience were noted between those diagnosed across various pathways; screen-diagnosed patients were less likely to report negative experiences than those diagnosed through emergency presentation [34]. Therefore, important differences may also be apparent after treatment and have implications for support through survivorship. In line with the common sense model [35], by understanding how colorectal cancer survivors make sense of their illness experience and current health status and manage future health threats, we are in a better position to support those who receive a screen diagnosis of colorectal cancer and enter an ongoing period of survivorship.

### 4.1. Limitations

Each interview describes a retrospective account of the individual's bowel cancer screening experience and could therefore be considered vulnerable to recall error and biased sampling. However, the biographical narrative interview method used encouraged a rich account of the subjective experience of receiving a diagnosis of cancer via screening, and therefore the focus is on participants' subjective experience rather than on accurate recall of events [28]. Furthermore, the phenomenological approach encourages a small and purposive sampling technique to enable in-depth idiographic analysis of interview data.

Limitations existed within the study sample, including heterogeneity of the sample which included participants in varying stages of their recovery (ranging from 1 to 11 years postdiagnosis). Nevertheless, all participants experienced significant changes in identity and were able to vividly recollect the memorable experience of diagnosis and what this meant to them and continues to mean to them in life postdiagnosis. Furthermore, it should be acknowledged that people with other screen-detected cancers may experience similar reactions to their diagnosis, changes in identity, and general adjustments to life posttreatment; however, this was not a comparative but rather an explorative study and, therefore, conclusions can only be representative of the participants of this study. Furthermore, it is important to note that participants were recruited for the main purpose of providing their personal story of screening to help in the development of a narrative-based leaflet to supplement current information provided to CRC screening invitees and were members of the Beating Bowel Cancer (BBC) charity. Therefore, the sample was limited to people who had, to some degree, sought support from a charity network and were motivated to help increase uptake of the current screening programme.

## 5. Conclusion

This phenomenological study of five individuals diagnosed with colorectal cancer via the NHS BCSP has highlighted key psychological implications of screening.

A screened-detected cancer is often detected and treated at an early stage, suggesting greater chance of survival. Therefore, people who chose screening are likely surviving longer after cancer than those diagnosed following symptomatic presentation. Understanding the long-term impact of a screen-detected diagnosis is instrumental to the optimisation of patient support through treatment and beyond. The complexity of the survivorship experience highlights the need for healthcare professionals to be aware that there is the potential for distress and anxiety even in periods of remission and that patient may need support to find ways forward. Future work could more directly consider comparisons with individuals in a post-treatment phase following a symptomatic diagnosis to assess if and when different supports are needed across the two patient groups.

## Figures and Tables

**Table 1:** Participant characteristics.

Participant (pseudonym)	Gender	Age at the time of interview	Marital status	Time since diagnosis (in years)
Simon	Male	68	Married	3
Celia	Female	64	Widowed	4
Bill	Male	73	Married	11
Helen	Female	63	Married	1
Pauline	Female	71	Married	2

## Data Availability

Anonymised interview transcripts are available from the corresponding author upon request.
